# Analysis of stress tolerance, competitive-anxiety, heart rate variability and salivary cortisol during successive matches in male futsal players

**DOI:** 10.1186/s13102-022-00582-3

**Published:** 2022-11-01

**Authors:** Bruno Laerte Lopes Ribeiro, Nicole Leite Galvão-Coelho, Raíssa Nóbrega Almeida, Gustavo Zampier dos Santos Lima, Leonardo de Sousa Fortes, Arnaldo Luis Mortatti

**Affiliations:** 1 Department of Physical Education, Federal University of Rio Grande do Norte – UFRN, Av. Senador Salgado Filho, 3000 – Lagoa Nova, Natal, RN CEP 59090-315 Brazil; 2 Clinical and Toxicological Analysis Department, Federal University of Rio Grande do Norte, Natal, Brazil; 3 School of Science and Technology, Federal University of Rio Grande do Norte, Natal, Brazil; 4 Department of Physical Education, Federal University of Paraíba - UFPB, João Pessoa, Brazil

**Keywords:** Endocrine responses, Monitoring competitive load, Rating of perceived exertion, Pre-competitive anxiety

## Abstract

**Background:**

This study aimed to compare the stress tolerance, competitive anxiety, heart rate variability and salivary cortisol before and during successive futsal competitive matches (3 matches in 4 days) in young male futsal players.

**Methods:**

10 young male futsal players (16.9 ± 0.7 age; 71.0 ± 5.1 kg; 174.9 ± 4.3 cm) were monitored during one training session and across a competitive period with 3 successive matches. External load was determined by the PlayerLoad method, while session rating of perceived exertion was used to calculate the internal training and competitive load. The stress tolerance was examined using Daily Analysis of Life Demand in Athletes questionnaire and the Competitive State Anxiety Inventory was used to analyze the competitive anxiety. The Time and frequency monitoring parameters were used to analyze the vagal cardiac autonomic marker. sC was analyzed using enzyme-linked immunosorbent assay.

**Results:**

A generalized estimating equation showed a significant difference for PlayerLoad from M1 to TS, M2 and M3, from M2 to M3 (*p* < 0.05), and for session rating of perceived exertion from M1 to Ts and M3 (*p* < 0.05). A difference for sources [χ^2^_(3)_ = 1.481, *p* = 0.68] or symptoms [χ^2^_(3)_ = 3.893, *p* = 0.27] was not found. There was no significant difference in any of the competitive anxiety [cognitive anxiety (F _(1.644; 14.799)_ = 4.6, *p* = 0.73, ŋ^2^* p* = 0.28), somatic anxiety (F _(2,09; 18,85)_ = 26.07 p = 0.057; ŋ^2^_p_ = 0.27) or self-confidence (F_(2.07; 18.85)_ = 15.875 p = 0.152; ŋ^2^_p_ = 0.18)] domains. The HRV parameters (time domain and frequency) and Salivary Cortisol (sC) (χ^2^_(3)_ = 4.320 p = 0.229) did not significantly change during the successive matches.

**Conclusion:**

The competitive scenario in which the players were evaluated did not significantly modify the stress tolerance, or the athletes’ state of anxiety, which in turn was not able to promote changes in the cardiac vagal modulation or in the sC levels before the matches.

## Background

The participation of young people in sports competitions has increased worldwide [1], however the stressors generated by training and competitions can lead to a psychological challenge, which in turn can be an influencing factor in sports performance and in the health of this population [2]. Thus, understanding that several factors can influence the stress caused by competition in basic categories such as pressure and challenges to win, poor coaching relationships and pressure by parents can negatively influence in the performance of athletes [2, 3]. In addition, stress is an important anxiety modulator, which is an important factor influencing competitive performance [4].

Thus, analyzing pre-competitive anxiety is an important topic in understanding how the competitive process can affect the emotional state of athletes, promoting physiological changes which can influence sports performance [5–7]. Among these changes, competitive stress can lead to changes in the cardiac autonomic nervous system as evidenced by a reduction in vagal tone, increased sympathetic activity and reduced parasympathetic activity in athletes with greater anxious behavior; this condition was observed in BMX athletes exposed to an official competition, in which the indexes related to heart rate variability (time and frequency domain) decreased, showing decreased vagal tone [8]. Moreover, inherent factors in the competition such as the opponent level can directly influence the anxiety state and HRV (time-domain (RR intervals, RMSSD) and non-linear parameter (SD1)) of athletes, even in team sports [9].

Alteration in the hypothalamic–pituitary–adrenal axis activity may also be related to the anxiety levels experienced by athletes, as cortisol levels are increased due to situations such as physical stress and sports competition [10, 11]. This condition was observed in the study by ^5^, in which judo athletes with higher cognitive anxiety levels showed higher salivary cortisol levels immediately pre- and post-competition compared to baseline values (day of rest). Arruda et al. [12] demonstrated an increase of pre-competition cortisol in elite basketball players according to the opponent level.

Competitions in the base categories in team sports such as futsal can involve very congested schedules, with successive matches being played with short recovery periods between them [10, 11]. This scenario has proven to be an important psychophysiological overload factor in youth players [13, 14].

Therefore, it seems necessary to analyze whether the stress levels and state of anxiety in young players due to the psychophysiological demands generated from performing successive matches may impact the pre-competitive anxiety state in youth players in a school-based situation. In view of this, the main objective of this study was to evaluate the stress tolerance and competitive anxiety in Under-17 male futsal players in a competition with successive matches at the school level. The study hypothesis was that the stress and anxiety levels do increase, in turn leading to a decrease in the HRV and an increase in salivary cortisol levels due to the competition of successive matches.

## Methods

### Subjects

A total of 10 male futsal players in the under 18 category (age: 16.9 ± 0.7 years, body mass: 71.0 ± 5.1 kg and height:174.9 ± 4.3 cm) were recruited to participate in the study. All participants were scheduled to participate in a school competition. The players trained twice a week divided into four stages: Warm-up, technical training (futsal fundamentals), tactical training (rehearsed moves and specific game situations) and finally the collective training (simulation of the matches). All athletes participating in the analysis had more than two years of systematic training in competitive futsal.

## Measures

### Importance and difficulty of the matches

**Fig. 1:**
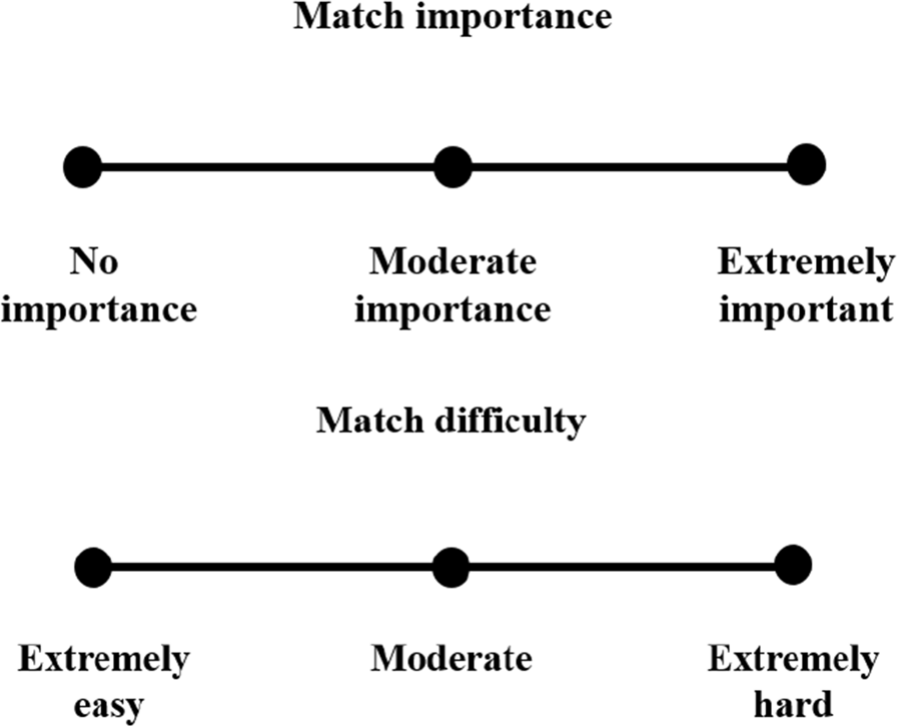
**A** Visual analog scale to match importance **B** visual analog scale to match difficulty

The coach was asked about the importance and difficulty of the matches performed. Thus, two visual analog scales were used for this evaluation, which indicated values of “no importance”, “moderate importance” and “extremely important”, regarding the importance of the matches. Regarding the difficulty analysis of the matches, the scale presented the options “extremely easy”, “moderate difficulty” and “extremely hard”. A moderate importance was reported in match 1, “low” (match 2) and “somewhat important” in match 3. Regarding the degree of difficulty, “moderate difficulty” was reported in match 1 and “low difficulty” in match 2 and 3 (Fig. 1).

### External load (EL)

The external training and match load were measured using the PlayerLoad method. This is an accelerometric measure which considers the activity time of each athlete and the direction change rate of the axes (x, y and z) [15]. The data for the three axes are integrated into the formula √ [(xn—xn—1) 2 + (yn—yn—1) 2 + (zn—zn-1) 2] in which the resulting values ​​are presented according to the duration of training sessions (PlayerLoad Total—PLT) and the average value per minute of activity (PlayerLoad per minute—PL / min), expressed in arbitrary units (au).

Accelerometers (Actigraph® GT3X, Pensacola, FL, USA) were used in monitoring the training sessions. The accelerometers were fixed on the individual’s right iliac crest with an elastic tape of adjustable size. The sampling rate was 100 Hz. The raw accelerometer data were analyzed in Matlab® Software (Version 9.3). The data analysis related to the external workload of the training week was performed through the average between the two weekly sessions.

### Perceived effort

The internal training and match load were determined by the Session Rating of Perceived Exertion (sRPE) using the Borg CR-10 scale adapted by Foster et al. [16]. The scale extremes were clarified to obtain better validity in the response, in which the zero anchor represents “rest” and ten which represents “maximum”. The individuals were encouraged to answer the question “How was your training/game?” at approximately 20 min after training/competition by choosing a descriptor (e.g., easy, difficult, maximum) and associating it with a numeric value. The data analysis referring to the RPE in the training week was performed through the average between the two weekly sessions.

### Stress tolerance

The Daily Analysis of Life Demand in Athletes (DALDA) questionnaire [17] was filled out at the end of week 2 and before each match. The DALDA questionnaire is divided into Part A (sources of stress) and Part B (symptoms of stress). The possible answers for each item are: “better than normal,” “normal,” and “worse than normal” and different scores were attributed for each concept: 1 for “worse than normal,” 2 for “normal” and 3 for “better than normal” [18]. A stress tolerance score was obtained through the sum of these values indicated by the individual. Higher scores indicate more tolerance to stress and vice-versa. Part A and part B of DALDA were analyzed and presented separately. Cronbach’s α for our sample was α = 0,5 for sources (Part A) and α = 0,8 for stress symptoms (Part B).

### Competitive state anxiety inventory-2 revised (CSAI-2R)

The Brazilian version of CSAI-2R [19] was used to assess the level of competitive anxiety in athletes. The questionnaire consists of 16 items which assess the athlete’s state of anxiety, subdivided into somatic, cognitive and self-confidence of the evaluated athletes. The scale score is measured by adding the values obtained in each answer, divided by the number of items in each sub-scale. The CSAI-2R is organized on a Likert scale, in which “1” represents nothing and “4” represents a lot. The higher the score, the higher the individual’s level of anxiety. Cronbach’s α for our sample was α = 0.55 for cognitive anxiety, α = 0.76 for somatic anxiety and α = 0.83 for self-confidence.

### Heart rate variability (HRV)

The RR intervals were obtained using a heart rate monitor with a telemetry signal (Polar® H10 with Bluetooth, KEMPELE, Finland) and an accelerometer (Actigraph® GT3X, PENSACOLA, FL, USA) with 100 Hz sampling. The RR intervals were recorded for five minutes with the athlete seated at rest in a room free from external noise. The parameters recommended by the Task Force [20] were used during the HRV collection. RR values were transferred to the Actilife software program (Actigraph® GT3X, PENSACOLA, FL, USA) and later exported to the Kubios® V2 software program (Polar Kubios V2, Kuppo, Finland), in which the final two minutes of the 5 min recorded were analyzed during data collection. The (RMSSD) mean difference between the squares of successive normal intervals and their logarithmic function (LnRMSSD) were performed to avoid outliers and simplify the analyzes.

### Saliva collection and assessment

The saliva samples were obtained in a standardized manner. Participants let the saliva drain into a suitable plastic container for the next 5 min in accordance with previous studies [13, 14]. The samples were then stored on dry ice, remaining during transport to the laboratory and transferred to the freezer (-80 °C). The tubes containing the saliva were centrifuged to separate mucus and cells. Supernatant was used in the analysis. The salivary cortisol was quantified using the ELISA method (ELX 800VV—Universal Microplate Reader, Bio-TeK instruments, USA). All samples were evaluated in duplicate and the mean absorbance of the two values ​​were used as the representative value. The average intra-assay coefficient of variation for the cortisol assays was less than 8%.

## Procedures

The players were followed for four weeks 3 training weeks (only the training week 2 was retained for analysis) and 1 week of competition (3 official matches). The competition is considered one of the most important within the school competition calendar and has many participating teams (92 teams). The competition was divided into two stages. The first stage was qualifying, in which groups were formed consisting of three teams which played against each other. The best two teams were qualified for the next stage, (knockout stage). The reference team won both matches 1 and 2 (7 × 1 and 6 × 0 respectively) of the group stage and was defeated (3 × 2) in the third match (knockout stage).

The study design was organized as follows: *Week 1 (Familiarization):* Familiarization with the instruments: use of accelerometers and cardiofrequency meter, rating of perceived exertion scale, as well as the DALDA and CSAI-2R questionnaires. *Week 2* (baseline week): The players were evaluated during a typical preparation week, two weeks before the start of the competition, with two weekly training sessions (Tuesday and Thursday), lasting for ~ 90 min. The HRV and saliva samples were collected before and sRPE after the first- and second-day training. CSAI-2R and DALDA questionnaires were filled in during rest on Friday. *Week 3 (tapering):* the players only participated in technical and tactical training sessions during the third week, characterized as “tapering phase”*; Week 4 (Competition):* The athletes performed three matches in four days. The first two matches had a 24-h interval between them and the interval between the second and third matches was approximately 48 h. The assessed matches lasted 80 min (2 × 40 min, with 10-min interval between halves). Salivary sampling and HRV was undertaken 60 min before each match and then CSAI-2R was filled out. The DALDA questionnaire was filled out after the matches and then the sRPE was reported (~ 20 min after each match).

### Statistical analyses

The Shapiro–Wilk test (*p* ≥ 0.05) was used to assess the data normality. Repeated measures one-way ANOVA analyzed the effect of time on the variables of CSAI-2R, and HRV variables (RR, PNN50, LF, HF), and sphericity was observed through the Mauchly test. Furthermore, the Greenhouse-Geiser correction was used if necessary. Bonferroni Post-hoc was used to identify pairwise comparisons. The effect size of the variables related to anxiety was assessed using the partial eta squared (η^2^), in which the values ​​considered were based on the criteria of Hopkins [21]. Friedman’s non-parametric test was used regarding the values of LnRMSSD, LnSDNN, LF/HF and sC, as well as the sources and symptoms of stress (DALDA). The sRPE analyzes and the PlayerLoad were performed using a Generalized Estimation Equation (GEE) test with Bonferroni’s post-hoc analysis. Cronbach’s Alpha was used for the analysis of internal consistency between the subscales of the CSAI-2R and DALDA questionnaires [22]. A *p*-value < 0.05 was considered significant for all analyses. The statistical package SPSS 25.0 for Windows was used for statistical analysis.

## Results

**Fig. 2:**
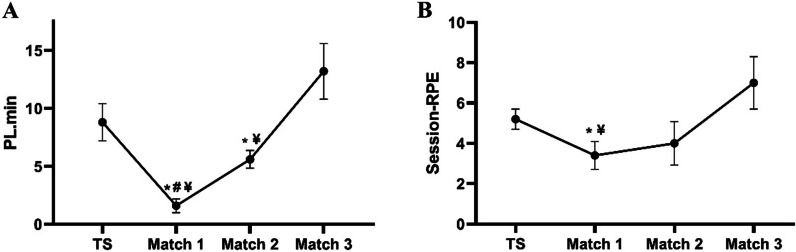
**A** PlayerLoad (PL/min) and **B** ratings of perceived exertion (session-RPE) for training day and each soccer match. Data presented as mean $\pm$ SD. *Significantly different to TS (*p* < 0.05). ^#^Significantly different to Match 2 (*p* < 0.05). ^¥^Significantly different to Match 3 (*p* < 0.05)

The PlayerLoad demonstrated significant difference between the four time-points [W_(3)_ = 112.4, *p* ≤ 0.001]. The values of PlayerLoad (PL/min) decreased from TS to match 1 (CI _95%_ =—10.4 – -4.0; *p* = 0.001) and increased from match 1 to match 2 (CI _95%_ = 2.6 – 5.5; *p* = 0.001) and match 3 (CI _95%_ = 6.9 – 16.3; *p* = 0.001). Match 2 showed a lower PlayerLoad (CI_95%_ = -6.3 – -0.04; *p* = 0.047) compared to TS and match 3 (CI_95%_ = 2.5 – 12.5; *p* = 0.003). The sRPE values of training and matches were significantly different between the time-points, W_(3)_ = 16.329, *p* = 0.001]. The sRPE values decreased from TS to match 1 (CI_95%_ = 0.3 – 3.2; *p* = 0.008) and increased from match 1 to match 3 (CI_95% =_ -6.7 – -0.3; *p* = 0.019) (Fig. 2).

There were no significant differences for sources (part A) [χ^2^_(3)_ = 1.481. *p* = 0.68] or symptoms (part B) [ χ^2^_(3)_ = 3.893, *p* = 0.27] of stress between the time-points (Table 1).

**Table 1:** Sources (Part A) and symptoms (Part B) of stress between training session (TS) and the matches. Values are expressed as median, 25th, and 75th percentiles

	TS	Match 1	Match 2	Match 3	*p*
Part A	17.0 (17.0; 18.2)	17.5 (17.0; 18.5)	17.5 (17.0; 18.5)	18.0 (16.75; 18.0)	0.68
Part B	52.0 (46.7; 59.0)	50.5 (49.7; 54.5)	50.5 (47.5; 53.7)	49.5 (45.5; 53.0)	0.27

There were no significant differences for cognitive anxiety [F_(1,644; 14,799)_ = 4.6, *p* = 0.73, ŋ^2^ = 0.28 (small)], somatic anxiety [F_(2.09; 18.85)_ = 26.07, *p* = 0.057, ŋ^2^ = 0.26] or self-confidence [F_(2.07; 18.85)_ = 15.875, *p* = 0.15, ŋ^2^ = 0.18 (small)] between the four time-points (Table 2).

**Table 2:** Values of somatic anxiety, cognitive anxiety, and self-confidence between training session (TS) and the matches. Values are expressed as Mean, ± SD

	TS	Match 1	Match 2	Match 3	ES	*p*
Cognitive anxiety	10.3 ± 1.0	10.8 ± 0.8	10.5 ± 1.0	11. 2 ± 0.9	0.28	0.73
Somatic anxiety	8.8 ± 0.9	11.0 ± 1.3	9.9 ± 1.1	10.4 ± 1.0	0.26	0.057
Self confidence	15.1 ± 0.4	14.5 ± 0.6	16.2 ± 07	14.9 ± 0.8	0.18	0.15

In relation to the HRV, the LnRMSSD values did not differ significantly between the moments of analysis [χ^2^_(3)_ = 3.360, *p* = 0.33], LnSDNN values also showed no differences in the observed moments [χ^2^_(3)_ = 3.727, *p* = 0.29] and [χ^2^_(3)_ = 1.560, *p* = 0.668]. Likewise, there was no difference between the four time-points for the sC values [χ^2^_(3)_ = 4.320 *p* = 0.29] (Table 3).

**Table 3:** Heart rate variability values (LnRMSSD, LnSDNN and LF/HF) and salivary cortisol between training session (TS) and matches. Values are expressed as medians (25th—75th percentiles)

	TS	Match 1	Match 2	Match 3	*p*
LnRMSSD	3.03 (2.93; 3.14)	3.15 (2.98; 3.34)	3.14 (2.71; 3.33)	3.08 (2.81; 3.34)	0.33
LnSDNN	3.81 (3.37;3.81)	3.36 (3.19;3.36)	3.29 (3.13;3.29)	3.53 (3.30;3.53)	0.29
LF/HF	2.70 (1.47; 5.27)	1.85 (1.10; 4.50)	2.60 (1.35; 4.40)	1.55 (0.70; 6.07)	0.66
Salivary cortisol (ng/mL)	2.32 (1.65; 2.96)	3.50 (1.84; 8.85)	3.16 (2.03; 3.86)	3.18 (2.05: 3.71)	0.29

LnRMSSD: Natural Logarithm the square root of the mean of the squares of the successive differences of the interval; LnSDNN: Natural Logarithm standard deviation of all normal RR intervals recorded in a time interval; LF/HF: Low frequency (LF) ratio (ms2) / High frequency (HF)

Still in relation to the HRV values, the RR [F_(1,635; 14,718)_ = 0.106, *p* = 0.863, ŋ^2^ = 0.012 (small)], pNN50 [F_(3;27)_ = 0.980, *p* = 0.417, ŋ^2^ = 0.098 (small)], LF [F_(3;27)_ = 0.569, *p* = 0.640, ŋ^2^ = 0.059 (small)] and HF [F_(3;27)_ = 0.536, *p* = 0.661, ŋ^2^ = 0.56 (small)]. (Table 4).

**Table 4:** Values of RR, PNN50, LF, HF and LF/HF between training session (TS) and the matches. Values are expressed as Mean ± SD

	TS	Match 1	Match 2	Match 3	Effect size	*P *value
RR	706.7 ± 49.2	698.9 ± 133.3	700.0 ± 80.5	719.8 ± 76.7	0.012	0.863
pNN50	2.4 ± 1.6	4.73 ± 3.7	3.7 ± 3.1	3.7 ± 2.9	0.09	0.417
LF	67.3 ± 14.2	63.8 ± 22.4	69.9 ± 14.2	60.2 ± 20.7	0.06	0.640
HF	33.9 ± 14.5	35.8 ± 22.1	29.9 ± 14.0	39.5 ± 20.5	0.05	0.661

RR: Time between intervals R-R; pNN50: Percentage of intervals > 50 ms different from the previous interval; LF: Low frequency; HF: High Frequency

## Discussion

The study analyzed the possible changes in the competitive-anxiety, HRV, and sC of a competition with successive matches in young school-level futsal players. The main findings were: (i) there was no change in the state of somatic anxiety, cognitive anxiety, or self-confidence of the participants throughout the competition; (ii) there was no change in the HRV levels or sC levels during the competition and when compared to the training week. Therefore, the study hypothesis that successive matches could affect anxiety and therefore that this could influence autonomic and endocrine modulation was not confirmed.

The PlayerLoad and sRPE measures of the training session (baseline) and of the three matches were performed to determine the magnitude of the loads imposed on the young players, since the workloads may have a direct implication in the psychophysiological response [23]. The PlayerLoad observed in the matches 1 and 2 were lower than TS, while the match 3 and TS values were similar. The PlayerLoad and sRPE demonstrated that the intensity of the matches was not greater than those experienced in the TS. In addition, it is worth mentioning that there were substitutions during matches 1 and 2, and all players played the matches; however, only 5 players were analyzed for internal and external match load in the match 3, in which there was no substitution. This fact may respond to the increase in external and internal match load of the players. the defeat (and elimination from the competition) in the match 3 can have influenced the increase in sRPE since this condition could be related to the importance of the match [24]. When analyzing the data of the present study, it can be observed that the report presented by the staffing coach points out that match 3 was the most important in relation to match 1 and 2.

Regarding ST, it was shown that there were no changes in the sources and symptoms throughout the analysis period, suggesting that the players were able to adequately deal with the stress imposed by successive matches. This result is in accordance with the study of Pinto et al. [25] which showed no change in ST during four basketball matches in three consecutive days in youth players. Freitas et al. [23] also found no differences in stress tolerance during 2-week overload followed by a 1-week taper during a pre-season. Furthermore, the psychological responses to stress seem to be associated with the increase in workloads imposed on athletes [26], which can be an explanation for our results since the athletes were below the usual training loads in the first two matches. Thus, it is understood that the match loads were not sufficient to alter the stress tolerance of the athletes.

The somatic and cognitive anxiety levels did not differ from the TS values, even considering the importance of the competition. This condition is like the results found by Hoover et al. [27] who assessed the state of somatic anxiety of 12 basketball players during three matches with different contexts (friendly and competitive matches) and found no differences in somatic anxiety levels. Another study in judo athletes demonstrated that the state of somatic and cognitive anxiety was higher according to the degree of importance of the competition ^5^.

It is important to note that the self-confidence values also did not differ between the moments, which can confirm that the competitive context combined with the successive match was not sufficient to produce psychological disturbances which could alter the state of competitive anxiety of young players. However, it was expected that the degree of importance before the third match due to the eliminatory character, and the possible cumulative fatigue effect generated by the successive matches would cause changes to occur in the competitive anxiety. It is possible that the two wins in the initial matches might have influenced the cognitive anxiety, somatic anxiety, and self-confidence scores in the knockout match.

Regarding the HRV, the stress generated by matches did not influence the autonomic modulation when compared to the TS. However, a study with BMX athletes in a simulated competition in one day of training and two days of an official competition, demonstrated that the official competition caused a reduction in the HRV in both, time, and frequency domains [8]. Although the competition in the present study was considered the most important in the school calendar, this degree of importance and match difficulty was possibly not high enough to alter the HRV at rest. This result was consistent with the results of the state of pre-competitive anxiety (somatic and cognitive), corroborating with the catastrophe theory and the multidimensional theory that demonstrate the need for an increase in anxiety, mainly somatic anxiety so that there is a stimulus for the physiological responses [28].

The sC values pre matches, were similar during the four analysis moments. This result corroborates with Moreira et al. [13], who did not find changes in the cortisol levels of 16 adolescent soccer players in seven successive matches for seven days. Although the authors did not address the aspects related to anxiety, it can be noted that there is no change in sC in youth soccer players, even with successive matches. However, the sC values seem to be in line with the results referring to somatic and cognitive anxiety which did not differ between the matches, not even in the third match (the knockout stage), and therefore the team to be faced was a higher level than previously faced in the classification phase. These results differ from the study by Arruda et al. [10], who demonstrated that pre-match salivary cortisol increased in basketball players according to the increase in cognitive and somatic anxiety in the games considered to be against the highest technical level of opponents.

Integrating the results of the variables analyzed in our study shows a coherence between the variables of physiological stress (HRV and salivary cortisol) with the stress tolerance and the anxiety state levels of young soccer players. These results confirm the importance of monitoring the workload during competitions and how the level of competition can influence the anxiety response of athletes.

## Conclusion

In conclusion, the results suggest that the competitive scenario in which the players were evaluated did not significantly modify the stress tolerance or the state of anxiety (somatic, cognitive and self-confidence), which in turn was not able to promote changes in the cardiac vagal modulation or in the sC levels before the matches.

## Data Availability

https://data.mendeley.com/datasets/nvbbg8f6x4/draft?a=89f1a01b-1403-45e4-8b76-a4a590aeae8f

## Abbreviations

HRV: Heart rate variability
sC: Salivary cortisol
TS: Training session
EL: External load
sRPE: Session rating of perceived exertion
DALDA: Daily analysis of life demand in athletes questionnaire
CSAI-2R: Competitive state anxiety inventory
GEE: Generalized estimating equation
